# The majority of genes in the pathogenic *Neisseria *species are present in non-pathogenic *Neisseria lactamica*, including those designated as virulence genes: response

**DOI:** 10.1186/1471-2164-7-129

**Published:** 2006-05-30

**Authors:** Richard Stabler, Jason Hinds

**Affiliations:** 1 Department of Infectious & Tropical Diseases, London, School of Hygiene and Tropical Medicine, London WC1E 7HT, UK; 2 Bacterial Microarray Group, Division of Cellular & Molecular Medicine, St George's, University of London, London SW17 0RE, UK

## Abstract

A response to Snyder LA, Saunders NJ: **The majority of genes in the pathogenic *Neisseria species *are present in non-pathogenic *Neisseria lactamica*, including those designated as virulence genes. ***BMC Genomics *2006, **7**:128.

## Background

The publication of a microarray analysis of selected *Neisseria lactamica *strains by Snyder and Saunders [1] has raised some interesting questions regarding the distribution of genes between pathogenic *Neisseria *species and the non-pathogenic *N. lactamica*, especially with regard to previously reported "virulence genes." This paper has also highlighted the difficulties in comparing microarray data from complementary yet distinct investigations and places particular emphasis on discrepancies with one of our own publications [2]. This comparison has raised general issues for discussion and a number of points for clarification that form the basis of this response.

Initially, the two papers may appear to take similar microarray-based approaches to address the general subject of specific gene distribution between pathogenic and non-pathogenic *Neisseria *species. Indeed, there are a number of similarities in the practical methods used, as would be expected for any microarray-based analysis, which makes the reported discrepancies all the more surprising. However, there are also a number of important distinctions to be made between the two studies, primarily based on experimental design and data analysis, which we believe greatly influence the interpretation of the results.

## Experimental design

Snyder and Saunders determined gene presence in a collection of 13 *N. lactamica *strains, with a particular focus on genes previously described as virulence genes, and also reported the presence of a subset of genes in a range of commensal *Neisseria *species. The microarray analysis was performed using a pan-*Neisseria *microarray with standard labelling and hybridisation protocols. Each *N. lactamica *strain was directly compared to two other strains in a loop design that incorporated a dye-swap although data was analysed in a channel-independent intensity-based manner.

Stabler *et al*. determined gene presence or absence in a collection of 38 *Neisseria *strains, including both pathogenic species and non-pathogenic commensal species, to establish the phylogenetic relationship of strains that showed favourable correlation to other typing methods. Genes present in all 18 *N. meningitidis *serotype B strains or present in 7 commensal strains were identified to compare the distribution of these genes in pathogenic and non-pathogenic strains. It is important to note that this differs to the goal of this study suggested by Snyder and Saunders. The microarray analysis was performed using a pan-*Neisseria *microarray with standard labelling and hybridisation protocols. Each *Neisseria *strain was directly compared to *N. meningitidis *MC58 in a common reference design and data was analysed in a channel-associated ratio-based manner.

In terms of the microarray platforms used, there are insufficient differences to explain the reported discrepancies. Clearly, the microarrays utilised by the two studies were of independent design and construction so the gene representation, reporter element sequences and PCR product amplicons will differ. However, given the rigorous approaches to design and construction of both arrays, these important but subtle differences are unlikely to account for the level of discordance reported. Even the strikingly similar hybridisation and washing conditions should produce a comparable level of stringency, which would be one obvious methodological reason for such widespread disagreement in the presence/absence calling of genes.

The major difference in the experimental design of the two studies comes from the comparisons made in each study and how these comparisons were executed. By their very nature, microarray comparative genomics studies are always limited by the gene content represented on the arrays and by the number and representation of strains analysed. In both cases, the arrays were essentially limited in content to the sequenced pathogenic *Neisseria *and therefore any novel genes present only in *N. lactamica *or other commensal species were not surveyed. Likewise, the strains selected in each study were considered by the authors to be representative and suitable for the intended investigation. It is evident that the analysis of additional strains may affect conclusions; Snyder and Saunders noted this with the inclusion of another *N. lactamica *strain that was not part of their initial MLST set and accounted for 72% of the strain specific genes they reported. However, the distinct selection of strains for the two studies still fails to fully explain the scale of discrepancies reported by Snyder and Saunders. One point to note would be that no pathogenic strains were included in the comparison by Snyder and Saunders whereas Stabler *et al. *compared all strains directly to a pathogen, namely the *N. meningitidis *MC58 common reference. This is important to note with regard to the execution of the comparisons for the data analysis approaches discussed in the following section.

## Data analysis

When considering the above points, in balance with other reasonable explanations, it would appear that data analysis and subsequent interpretation represents the key area in which the two groups adopted fundamentally different approaches that may help to explain the discrepancies observed. Snyder and Saunders employed an intensity-based method that analysed the two channels of a two-colour microarray independently whereas Stabler *et al. *used a ratio-based method that analysed the two channels of a two-colour microarray in combination.

Snyder and Saunders used a metric (pON) that reflects the probability of there being a hybridisation signal for each spot in each channel independently. Whilst this seems a reasonable approach it takes no account of any relative intensity levels; a gene may be called present in two strains if the pON threshold is exceeded in both yet the relative intensity level may be significantly higher in one strain than the other. For example, for a pathogen gene that was highly divergent in *N. lactamica *there may still be a low but sufficient signal intensity above background to call the gene as present in *N. lactamica *using the pON metric, yet this level of hybridisation would be insignificant when compared to the signal intensity achieved with a pathogen; therefore a more accurate call for this gene should be absent or highly divergent rather than present. This also raises the issue that the PMT gain settings during scanning may have an impact on the number of genes passing the pON threshold and thus being assigned as present. If this were true then a normalisation strategy would need to be employed to account for this to ensure the definition of presence is consistent across different arrays and strains and is not dependent on scanning. Furthermore, the comparison of absolute intensities for different genes can be affected by factors such as the concentration, length, and Tm of reporter elements. The authors have essentially adopted a one-colour approach to the analysis of two-colour data, although generally some form of normalisation and an attempt to address the issue of relative intensity would be included. However, in their study relative intensities would not have been informative as no pathogens were included in the comparison.

Stabler *et al. *approached data analysis in a more common approach for two-colour data by first removing unreliable low intensity data based on image analysis QC flags, normalisation of ratio data to remove any systematic effects and applying ratio cutoffs to determine genes present or absent in comparison to the control strain hybridised to each array as a common reference. The GACK software used to set dynamic ratio cutoffs and make the 'present', 'divergent' or 'absent/highly divergent' calls was set at its most conservative, classified as 'present' if the estimated probability of presence (EPP) = 100% and 'absent/highly divergent' if EPP = 0% with 'divergent' genes between these two extremes. Snyder and Saunders wrongly indicate that the calls of absence by Stabler *et al. *were based purely on a lack of hybridisation; if this were the case then we would agree with the other possible technical reasons for this that they describe in detail. In actual fact, the calls of absence by Stabler *et al. *are based on the ratio of intensities for the test strain channel and the common reference strain control channel. Therefore, the call of absence depends on direct comparison to the positive signal intensity for the same spot which serves as an internal control. This presents one of the great strengths of two-colour analysis that helps to circumvent the highlighted problems of missing or poorly performing reporter elements as the same reporter is being used in each channel of the comparison.

These fundamental differences in data analysis present the most likely source of discrepancies between the two studies as all subsequent interpretations are based on the issue of gene presence in different strains. Snyder and Saunders were likely to overestimate gene presence and unable to discriminate conserved genes from highly divergent genes as relative intensities are not taken into account nor any direct comparison to pathogens. In contrast, Stabler *et al. *are likely to underestimate gene presence due to the perhaps overly strict criteria applied to gene selection, that is, the need for a 100% certainty of gene presence in every strain within a group, that would exclude any gene just missing these thresholds in a single strain. Given these two likely extremes it is not surprising that so many discrepancies were reported.

## Data interpretation

The final interpretation of data is clearly dependent on the methods used to determine gene presence as outlined above. Many of the discrepancies reported by Snyder and Saunders were related to comparison with the microarray data of Stabler *et al. *but also extended to other studies identifying "virulence genes" or pathogen-specific genes. Whilst we want to avoid an extended reanalysis and interrogation of both datasets, Snyder and Saunders make critical misrepresentations of Stabler *et al. *data that need to be addressed in more general terms.

One section discussed in detail by Snyder and Saunders relates to the genes presented by Stabler *et al. *in Table 3. This list of genes was produced by identifying genes present in all *N. meningitidis *serotype B strains and excluding genes classified as absent in all commensal strains analysed. The extremely stringent method employed was purposefully chosen due to the relatively low numbers of strains compared in this study and the desire to avoid any false positives. Whilst there is agreement with Snyder and Saunders on the known strain-specific genes, reflecting confirmed gene absence, there is less agreement on a significant number of genes that will most likely differ in the degree of sequence divergence and thus relate to the methods used to determine gene presence. Confidence in our own data comes from the fact that the relative intensities of these genes are in the order of 500 times lower in the commensals than the pathogens when compared to the common reference, suggesting these genes are highly divergent and thus likely to encode functionally distinct proteins.

Table 3 in Stabler *et al. *is also discussed in relation to the pathogen-specific genes reported by Snyder and Saunders in Table 3 as 5/6 genes were not reported by Stabler *et al. *However, it is incorrect and misleading to conclude that lack of genes in this list equates to presence in commensals as suggested. The reason for these genes not appearing in Stabler *et al. *Table 3 was due to them not meeting the strict criteria for inclusion rather than indicating presence in commensals. The data for these genes also demonstrates excellent differential distribution between pathogens and commensals and so is in agreement with the findings of Snyder and Saunders. This does highlight that perhaps the analysis stringency of Stabler *et al. *should be reduced to ensure that potential genes are not missed although this would increase the risk of more false positives.

## Summary

This response has hopefully highlighted some of the issues that influence the interpretation of microarray data from different groups. To a non-specialist reader this may seem bewildering but it is important to appreciate that the application of apparently similar experimental approaches can lead to very different conclusions and the need to understand the basis of this.
